# Report From the 6^th^ International Meeting on Bone Marrow Adiposity (BMA2020)

**DOI:** 10.3389/fendo.2021.712088

**Published:** 2021-07-16

**Authors:** Erica L. Scheller, Meghan E. McGee-Lawrence, Beata Lecka-Czernik

**Affiliations:** ^1^ Division of Bone and Mineral Diseases, Department of Medicine, Washington University, Saint Louis, MO, United States; ^2^ Department of Cellular Biology and Anatomy, Medical College of Georgia, Augusta University, Augusta, GA, United States; ^3^ Departments of Orthopaedic Surgery, Physiology and Pharmacology, Center for Diabetes and Endocrine Research, University of Toledo, Toledo, OH, United States

**Keywords:** bone marrow adiposity, bone marrow adipocyte, marrow fat, yellow marrow, bone metabolism, bone marrow adiposity society (BMAS)

## Abstract

The 6^th^ International Meeting on Bone Marrow Adiposity (BMA) entitled “Marrow Adiposity: Bone, Aging, and Beyond” (BMA2020) was held virtually on September 9^th^ and 10^th^, 2020. The mission of this meeting was to facilitate communication and collaboration among scientists from around the world who are interested in different aspects of bone marrow adiposity in health and disease. The BMA2020 meeting brought together 198 attendees from diverse research and clinical backgrounds spanning fields including bone biology, endocrinology, stem cell biology, metabolism, oncology, aging, and hematopoiesis. The congress featured an invited keynote address by Ormond MacDougald and ten invited speakers, in addition to 20 short talks, 35 posters, and several training and networking sessions. This report summarizes and highlights the scientific content of the meeting and the progress of the working groups of the BMA society (http://bma-society.org/).

## Introduction

**Figure 1:**
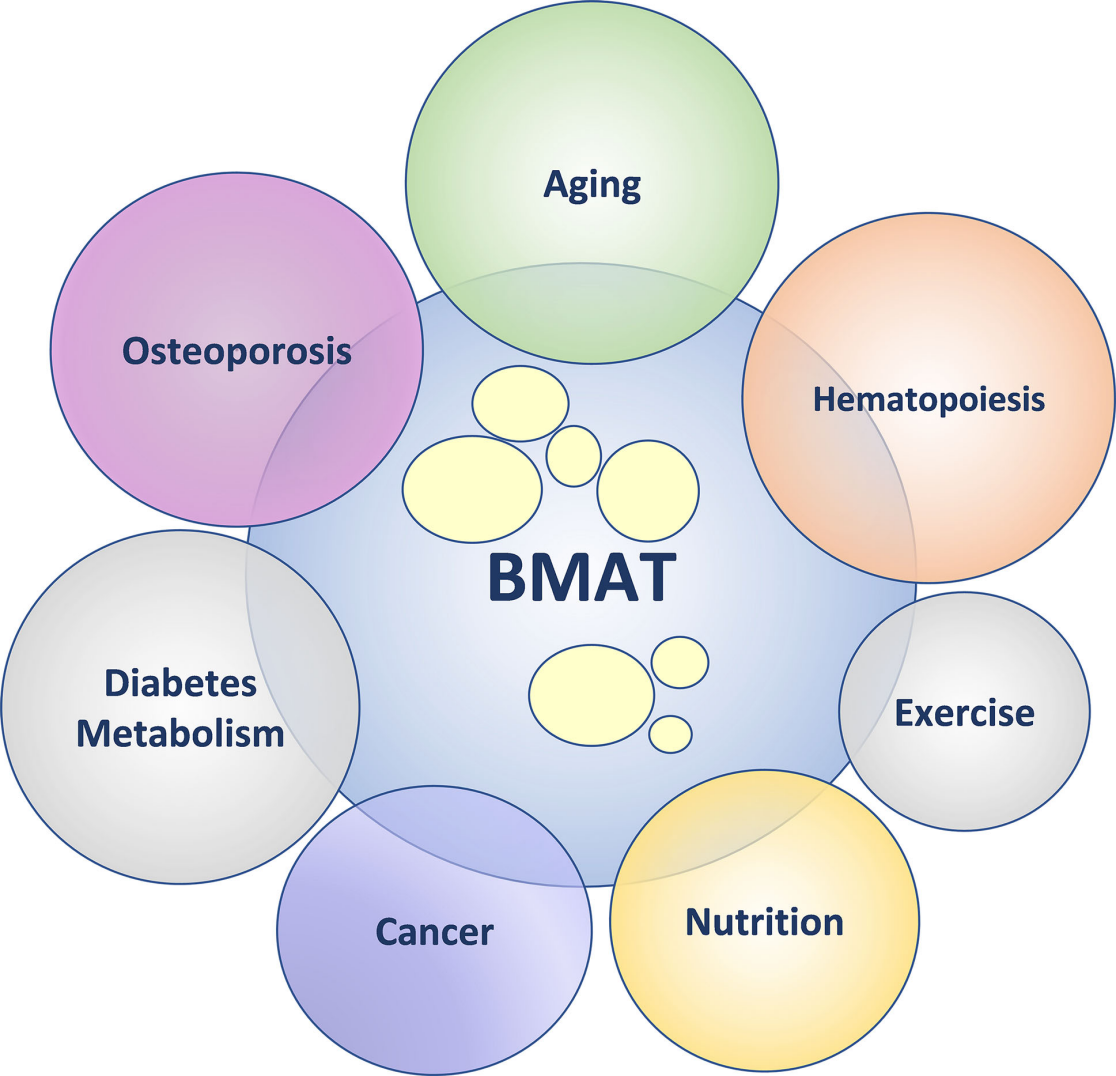
Bone marrow adipose tissue (BMAT) – friend or foe? In health, BMAT supports physiological homeostasis. In pathological states, BMAT augments dysregulation of physiological homeostasis. The diagram depicts the emerging topics within the field of BMAT research.

The primary goal of the 6^th^ international meeting on Bone Marrow Adiposity, “Marrow Adiposity: Bone, Aging, and Beyond” (BMA2020), was to provide a forum for basic, translational, and clinical scientists from around the world to discuss different aspects of bone marrow adiposity in health and disease. The Bone Marrow Adiposity Society (BMAS) conferences are greatly enhanced by the broad research and training backgrounds among conference organizers, speakers, and attendees that promote diverse perspectives and opportunities for collaboration. The field of bone marrow adipose tissue (BMAT) research is growing, which was reflected in the BMA2020 program. Besides a specific focus on aging and osteoporosis, the BMA2020 meeting program included sessions related to the role of BMAT in hematopoiesis, regulation of systemic energy metabolism, cancer development and metastasis, and response to environmental cues including nutrition and exercise (See **Figure 1**).

The BMA2020 meeting was hosted in partnership with the American Society for Bone and Mineral Research (ASBMR) and was co-organized by Drs. Erica Scheller (Washington University), Meghan McGee-Lawrence (Augusta University), and Beata Lecka-Czernik (The University of Toledo). BMA2020 was originally scheduled to take place in Seattle, Washington as the first BMA meeting in the United States. However, due to the global COVID-19 pandemic, the congress was instead hosted entirely online in a mixed live/asynchronous format to promote both networking and accessibility. With great thanks to the dedication of our BMAS members, both established and new, this meeting ended up being our largest event to date with 11 invited speakers, 55 submitted abstracts, and 198 registered attendees from 18 different countries around the world. The congress featured 53 sessions and sub-sessions, 35 posters, and 14 lab highlights that were viewed collectively more than 7,000 times. In direct evidence of the collaborative nature of the meeting, there were also 63 photos shared and 1,391 in-app messages sent between colleagues. In all, despite the change in format, the BMA2020 meeting succeeded in bringing together clinicians and scientists studying all aspects of bone marrow adiposity, contributing to the advancement of the field.

## Scientific Sessions

The scientific sessions at BMA2020 included bone marrow adipocyte (BMAd) dynamics and bone mass in aging, obesity and malnutrition, as well as BMAd progenitors and lineage tracing, metabolism, hematopoiesis, metastasis, leukemia and myeloma, endocrine regulation, exercise, and advanced methods of BMAd imaging and analysis, as discussed below. A full list of talks and speakers is available in **Supplemental Table 1**. Where available, references to recently published related manuscripts are provided below.

### Keynote Address

BMA2020 opened with a welcome address from the conference organizers, followed immediately by a keynote presentation by Ormond MacDougald (University of Michigan) entitled “Bone marrow adipose tissue biology, endocrine physiology and metabolism – unique and common traits with peripheral adipose tissue depots”. In his talk, Dr. MacDougald highlighted the basis of BMAd cell biology and physiology as grounded in the broader field of adipose tissue biology. He went on to discuss the nature of regulated and constitutive BMAT clusters (rBMAT and cBMAT, respectively), the discovery of which ultimately provided bases for in-depth analysis of the unique metabolic regulation of BMAT relative to other fat depots in the body (1). Thus, rBMAT located in proximal skeletal elements and closer to body core temperature is interspersed with hematopoietic, stromal and vascular cells and changes its volume in response to hormonal, nutritional and pharmacological challenges. In contrast, cBMAT, which is located distally and relatively further from the body core temperature, shares similar characteristics with white adipose tissue and is largely resistant to environmental stimuli (2). However, cBMAT volume in humans may change after bariatric surgery and in certain hematologic conditions (3). Dr. MacDougald also introduced the first animal model that specifically targets marrow adipocytes by expressing both adipocyte-targeted (adiponectin) and osteoblast-targeted (osterix) markers, a combination that is characteristic for BMAds. This conditional model may ultimately help the field to understand BMAT function in diverse settings of bone loss, as well as its contributions to systemic energy metabolism. In summary, this keynote presentation was a perfect introduction to the entire conference by highlighting the unique position of BMAT among other fat depots, which may explain its divergent functions in health and disease as explored in the subsequent sessions.

### Session I: Bone Marrow Adipose Tissue, Aging, and Skeletal Homeostasis

The first session featured an invited talk by Sundeep Khosla (Mayo Clinic) that covered aspects of BMAT, aging, senolytics and skeletal health. A key focus of his presentation was that cellular senescence may play a causal role in reducing osteoblastogenesis and increasing adipogenesis during the process of aging, suggesting that senolytic drugs have potential to target this aspect of skeletal aging biology (4). The invited talk was followed by four short talks from Anuj Sharma (Augusta University), Abhishek Chandra (Mayo Clinic), Thomas Ambrosi (Stanford University), and Vagelis Rinotas (Agricultural University of Athens) that covered topics including sex differences in marrow adiposity induced by glucocorticoid signaling, radiation-associated bone marrow adiposity, skeletal stem cell diversity, and the role of RANKL in development of BMAd expansion and osteoporosis. The session was moderated by Bram van der Eerden (Erasmus University Rotterdam) and Rosella Labella (Columbia University). The presentation content, and subsequent discussion, focused heavily on mechanisms of cross-talk between cells, and the influence of the bone marrow niche environment (particularly with aging) on mechanisms of increasing marrow adiposity. The first short talk highlighted the sexually dimorphic low bone mass and high BMAT phenotype of mice with conditional deletion of the glucocorticoid receptor in osteoprogenitor cells (5). The second talk demonstrated the time course of molecular events leading to enhanced marrow adiposity following irradiation of bone, and highlighted similarities in mechanisms of senescence between aging and irradiated bone that precede fatty infiltration of the bone marrow cavity (6). In the third talk, single cell RNA-sequencing was used to show alterations in the skeletal stem cell populations that promote a pro-inflammatory, pro-resorptive, pro-adipogenic, and anti-osteogenic environment within the bone marrow niche that could be pharmacologically targeted (7). The fourth and final short talk characterized marrow adiposity and BMSC differentiation patterns in two different RANKL transgenic mouse models and explored the molecular mechanisms underlying the observed phenotypes (8).

### Session II: Environmental Regulation of Bone and Marrow Adipose Tissue

The second session included two invited talks by Pouneh Fazeli (University of Pittsburgh) and Janet Rubin (University of North Carolina), followed by a short talk from Piotr Czernik (The University of Toledo). It was moderated by Anne Schafer (University of California San Francisco) and Rebecca Schill (University of Michigan). The session was concentrated on regulators of BMAT volume including nutrition, exercise and gut microbiota. Dr. Fazeli reviewed research focused on the paradox of increased BMAT volume in anorexia nervosa, a clinical condition of chronic undernutrition, with a take home message that BMAT serves different functions in states of nutrient sufficiency as compared to nutrient insufficiency (9). In addition, she emphasized the important function of BMAT in regulation of hematopoiesis by demonstrating that BMAT is inversely associated with white and red blood cell counts in premenopausal women, and by showing that in women with anorexia treated with transdermal estrogen, decreases in BMAT were significantly associated with increases in both red blood cells and hematocrit (10). Dr. Rubin focused her presentation on the effect of exercise on adaptive changes in bone, including decreases in BMAT volume and increases in osteogenesis. This presentation added new information to the ongoing discussion on sequestration of β-catenin and Wnt pathway activity in regards to the BMSC lineage commitment and identified EZH2 as key to preservation of BMSC multipotentiality *via* β‐catenin (11, 12). To conclude the session, a short talk by Piotr Czernik revealed that reconstitution of the gut microbiome in germ-free rats increased marrow adipocyte number and altered their cell size distribution toward smaller adipocytes. His work suggests that gut microbiota deliver powerful signals to the skeleton to regulate bone marrow adipocyte differentiation and function and that *de novo* expansion of small adipocytes synergizes with new bone formation in conditions of acute nutrient utilization in the gut (13).

### Session III: Endocrine Regulation of Bone Marrow Adipose Tissue in Health and Disease

Session III began with an invited talk by Clifford Rosen (Maine Medical Center Research Institute). This presentation focused on the role of hormones, especially parathyroid hormone, in the regulation of BMAT *via* mechanisms such as lineage recruitment and lipolysis (14, 15). The invited talk was followed by three short talks by Nikki Aaron (Columbia University), Sudipta Baroi (The University of Toledo), and Li Chen (Southern Denmark University) that covered topics including novel adipokines, osteocytic control of marrow adiposity *via* sclerostin production, and crosstalk between the immune system and bone. This session was moderated by Laura McCabe (Michigan State University) and Biagio Palmisano (Columbia University). The first short talk reported the role of the novel adipokine adipsin and its ability to activate the immune complement system in BMAT expansion promoted by caloric restriction or thiazolidinedione treatment (16). The second short talk described the role of PPARγ in osteocytes, showing that the sclerostin gene is regulated by PPARγ and that osteocyte-specific conditional knockout mice have a high bone mass and low BMAT phenotype (17). The third and final short talk in this session examined the regulatory role of the immune system complement factor H in bone and BMAT homeostasis (18).

### Session IV: Advanced Methods for Clinical and Pre-Clinical Assessment of Bone Marrow Adiposity and Skeletal Health

This methods-focused session started with two invited speakers, Gustavo Duque (University of Melbourne) and Greet Kerckhofs (Université Catholique de Louvain), followed by two short talks from Kisoo Pahk (Korea University Anam Hospital) and Josefine Tratwal (Ecole Polytechnique Fédérale de Lausanne). Session IV was moderated by Jean-Francois Budzik (Groupement des Hôpitaux de l’Institut Catholique de Lille) and Ahmed Al Saedi (University of Melbourne). Dr. Duque discussed secreted factors that couple bone marrow adipocytes to the surrounding skeletal cells and their implications for the pathophysiology of osteoporosis. In addition, he provided information about key technical approaches that can be used to study aspects of bone marrow adipocyte function and lipotoxicity both *in vitro* and *ex vivo* (19, 20). After this, Dr. Kerckhofs demonstrated how novel contrast agents such as polyoxometalate can be used to visualize and to quantify bone marrow adipocytes in three dimensions, in addition to relating this information to bone and vascular quantifications using high-resolution computed tomography (21). Next, Kisoo Pahk reported the use of 18F-FDG PET/CT for the evaluation of adipose tissue metabolic activity in patients with osteoporosis, demonstrating that metabolic activity of visceral *vs* subcutaneous adipose tissue is predictive of bone mineral density (22). Last, Josefine Tratwal gave an overview of a new injectable, three-dimensional tissue engineered model of bone marrow adipogenesis and hematopoiesis that can be used to accelerate studies on the regeneration of the bone marrow niche for treatment of blood and other marrow-related disorders (23).

### Session V: Bone Marrow Adipose Tissue, Cancer, and Hematopoiesis

The invited speakers for Session V were Olaia Naiveras (École Polytechnique Fédérale de Lausanne) and Izabela Podgorski (Wayne State University). Short talks were given by Emma Morris (University of Oxford), Sonia Severin (Inserm U1048 and Paul Sabatier University), and Mariah Farrell (Maine Medical Center Research Institute). The session was moderated by Michaela Reagan (Maine Medical Center Research Institute) and Josefine Tratwal (Ecole Polytechnique Fédérale de Lausanne). Session V focused on mechanisms by which BMAT regulates hematopoiesis and cancer progression. Olaia Naveiras summarized the effects of BMAT on hematopoiesis, including use of MarrowQuant, a new tool for quantification of bone marrow compartments in histologic sections (24). This presentation concluded that marrow adiposity correlates inversely with hematopoiesis in health and pathologic conditions, and that the degree of adipocyte maturation correlates inversely with the proliferation of hematopoietic precursors. In addition, Dr. Naiveras identified loss of SCF upon differentiation of BMAT progenitor cells as responsible for decreased support of hematopoiesis (25). After this, Izabela Podgorski showed that BMAT fuels and supports growth of prostate cancer metastasis to bone by serving as an abundant source of lipids and signaling molecules. She demonstrated that metastatic tumor cells engage in reciprocal interactions with bone marrow adipocytes to evade therapy and provided evidence that targeting IL-1β or lipolysis improves tumor cell response to anti-cancer therapy with docetaxel (26). Dr. Podgorski concluded her talk by stressing an importance of understanding the role of BMAT in tumor adaptation and survival in bone, as a tool to reveal novel, mechanistic targets for therapies for prostate and breast bone-metastatic diseases, which continue to be incurable (27). After this, Emma Morris discussed the effects of PPARγ agonist BAGDE on myeloma cells and bone marrow adipocytes (28), Sonia Severin showed a functional link between marrow adipocytes and the development of megakaryocytes (29), and Mariah Farrell revealed that bone marrow adipocytes support multiple myeloma drug resistance and induce adipocyte mimicry, increasing cell survival (30, 31). Overall, this session stressed an importance of understanding the role of BMAT in hematopoiesis and cancer cell survival in bone, while underscoring a prominent role of adipocytes in the regulation of different components of hematopoietic niche.

### Session VI: Bone Marrow Adipose Tissue Origins and Maturation

This session featured an invited talk by Moustapha Kassem (University of Southern Denmark) and three short talks by Russell Turner (Oregon State University), Leilei Zhong (University of Pennsylvania), and Xiao Zhang (Washington University). Session VI was moderated by Michaela Tencerova (University of Southern Denmark) and Sudipta Baroi (The University of Toledo). Dr. Kassem opened the session by reviewing the mechanisms underlying lineage commitment of multipotent bone marrow stromal (skeletal) stem cells to adipocytes with an emphasis on the role of KIAA1199 (32). Next, Dr. Turner presented data showing that c-kit expression in hematopoietic and mesenchymal cells contributes to the maturation of bone marrow adipocytes (33). After this, Leilei Zhong presented new data that defines a stromal/perivascular, adiponectin-expressing bone marrow adipocyte progenitor, termed the marrow adipogenic lineage precursor (MALP) and its role in the maintenance of the bone marrow vasculature (34, 35). Last, Xiao Zhang showed that a unique population of maladapted bone marrow adipocytes was retained in an otherwise ‘fat free’ lipodystrophic mouse, revealing the existence of a bone-specific, compensatory adipogenesis pathway that is activated in states of metabolic stress (36). Overall, this session contributed to our understanding of the unique cellular origins of bone marrow adipocytes during development and metabolic disease, in addition to providing new information about the molecular signals regulating the maturation of committed pre-adipocytes within the bone marrow.

### Session VII: Bone Marrow Adipocyte Differentiation, Metabolism and the Skeletal Niche

The final session started with an invited talk by Courtney Karner (University of Texas Southwestern Medical Center), followed by four short talks by Ziru Li (University of Michigan), Ayyoub Salmi (MABlab), Amit Chougule (University of Toledo), and Wei Yu (University of Pennsylvania). The session was moderated by Christophe Chauveau (MABLab) and Thomas Ambrosi (Stanford University). Dr. Karner presented on the role of glutamine metabolism in the fate specification of bone marrow adipocytes from mesenchymal precursor cells, providing support for a model by which inhibition of glutamine metabolism or glutathione biosynthesis increases oxidative stress and promotes adipocyte differentiation (37). After this, Dr. Li presented work in a conditional adipose triglyceride lipase knockout mouse demonstrating roles for bone marrow adipocyte lipolysis in states of nutrient deficiency. Next, Ayyoub Salmi presented evidence in support of the capacity for osteoblasts to transdifferentiate to adipocyte-like cells following co-culture with adipocytes, emphasizing the plasticity and intercellular communication of cell populations within bone (38). This was followed by a presentation from Amit Chougule showing that activation of PPARα in the osteocyte lineage suppresses bone marrow adipose tissue expansion in regions of hematopoietic marrow. Last, Wei Yu demonstrated that adiponectin-expressing MALP cells are a critical source of osteoclast-regulatory factor RANKL, contributing directly to osteoclast formation and bone turnover (35). The questions and discussion in this diverse session centered on Cre specificity and the use of Cre-dependent lineage tracing when compared to surface marker strategies for characterization of bone marrow adipocyte progenitors. In addition, the group emphasized the need to continue to work together to unify our models and definitions to promote the advancement of future work in the field.

## Posters

Posters consisted of a pre-recorded video presentation of up to 5-minutes in length. Virtual posters were highlighted in the exhibitor center and could be viewed asynchronously by congress attendees. A live poster session was also held on the first day of the conference where attendees and presenters could post and respond to questions in real time. The highlighted topics of the posters fell into several main categories including aging, BMAT dynamics and bone mass, endocrine regulation, imaging and advanced methods, metabolism, metastasis, myeloma and hematopoiesis, obesity and malnutrition, and progenitors and lineage tracing. A detailed list of posters and presenters is available in **Supplemental Table 2**.

## Networking and Professional Development

The Bone Marrow Adiposity Society is committed to fostering collaborations between members and to the development of trainees and fellows to support the growth and advancement of the field. Consistent with this mission, the BMA2020 meeting featured several trainee discussion sessions and networking events. All members were invited to participate in an open discussion with keynote speaker Dr. Ormond MacDougald, a question-and-answer session with leaders from the National Institutes of Health, and a BMA2020 ‘After Hours Trivia’ social event hosted by Don’t Tell Comedy. In addition, there were two trainee-specific sessions focused on topics including publishing, preprints, and open access (led by William Cawthorn, The University of Edinburgh) and planning for life and job opportunities after research training (led by Gina Woods, UCSD, Hai-Bin Ruan, University of Minnesota, and Christa Maes, KU Leuven). The meeting organizers would like to provide special thanks to Andrea Lovdel and Biagio Palmisano for their contributions to the development of the trainee-focused content and trivia event of the BMA2020 meeting.

## BMAS Working Groups

In addition to networking and scientific content, the BMA2020 symposium provided a forum for meetings of the six BMAS working groups (WG). Additional information on the activities of the working groups can also be obtained by following BMAS on twitter (@BMA_Society).

**WG1 – Nomenclature.** The nomenclature working group position paper reporting Standardized Nomenclature, Abbreviations, and Units for the Study of Bone Marrow Adiposity was published in 2020 (39). The current goal of WG1 is to promote the awareness and application of these guidelines among researchers, journal editors and publishers, and to continue monitoring the emergence of new terms and methods to ensure that the guidelines are updated as the field continues to develop.

**WG2 – Methodologies.** The goal of the methodologies working group is to encourage the use of standardized methodologies for the assessment of bone marrow adipocytes. The methodologies working group recently published a review on standardization in methodology for the study of bone marrow adiposity (40). After welcoming new members, the methodologies working group decided on new projects. In the coming years the group will focus on in-depth review and discussion of *in vivo* imaging methods and *in vitro* systems in the study of bone marrow adiposity, leading to future publications on the topic to promote the standard application of methods in the field.

**WG3 – Biobanking.** The aim of the biobanking working group is to establish the use of standardized protocols for the collection and storage of materials related to bone marrow adipocytes. WG3 is currently preparing a position paper on biobanking of BMAd-related material. This position paper will cover several aspects of biobanking including introduction to biobanking with a focus on BMAd-related material, patient information, pseudonymization and ethics, types of tissues/sources from which BMAd-related material can be obtained, and isolation protocols to collect bone marrow adipocytes and bone marrow mesenchymal stromal cells for future applications. With this, WG3 aims to reach clinicians and scientists involved in biobanking of BMAd-related material by providing considerations on the collection, storage and use of BMAd-samples.

**WG4 - Public Engagement.** The working group on public engagement focuses on ways to educate target groups outside the society, including other societies, patient organizations and the public. Since the start of the working group, fruitful collaborations have been set up with affiliating societies, such as ECTS and ASBMR. Finally, WG4 is involved in managing the social media (Twitter, LinkedIn) of BMAS.

**WG5 – Repositories.** The working group on repositories aims to identify currently existing repositories that contain expression data related to BMAd. Ultimately, this information can be distributed among BMAS members to gain access to the BMAd-related repositories for their research.

**WG6 – Sponsoring.** The aim of the sponsoring working group is to find partners in industry and academia to financially liaise with BMAS. This committee is tasked with developing a strategy to ensure stable sponsorships for BMAS.

## Awards

Awards were presented during the conclusion of the BMA2020 meeting for the highest scoring clinical research and basic/translational research short talks and poster presentations. In addition, to promote scientific discourse and interaction during the virtual meeting, awards were also presented for the “audience choice” best poster presentation, a social media-based photo contest, a community engagement award, and a trivia contest. For the abstract-based scientific awards, all abstract submissions underwent blinded review and scoring by three to five independent reviewers. Abstracts were categorized as “clinical research” or “basic/translational research”, and awards were administered proportionally to the volume of abstracts received in each category; seven such awards were presented for basic/translational research, and three for clinical science. For the short talk and poster presentation awards during the meeting, presentations were scored by representatives from the BMAS Scientific Board on the overall presentation quality, the speaker’s responses during the question and answer session, the impact of the data, and the overall communication of the message. A full list of scientific awards and recipients is provided in **Supplemental Table 3**.

## Concluding Remarks and Perspectives

BMA2020 was the 6^th^ International Meeting of the Bone Marrow Adiposity Society. This was an important forum for discussion within the diverse, growing field of bone marrow adiposity research. The success of the transition to a virtual format has prompted discussions regarding the future integration of both in-person and virtual events to support the attendance of those that are not able to travel to the on-site destination. Moving forward, the BMAS meetings will be held once every 2-years with the next BMA2022 meeting currently scheduled to take place in Greece under the direction of Eleni Douni. In addition to this, several trainees and members within BMAS have come together to organize a virtual training seminar entitled “BMAS Summer School 2021”. This training seminar will take place over three half-days in September of 2021 and feature topical lectures from international experts, skill-based workshops, group discussions, and presentations that will promote the career development of trainees in the field. In conclusion, we are thankful for the overall success of the BMA2020 symposia and the connections made between colleagues despite the global COVID-19 pandemic. Though bone marrow adipocytes are gaining increasing attention as an endocrine cell and key component of the skeletal niche, much remains to be discovered to harness their potential in anti-osteoporotic, metabolic, cancer, hematological, or regenerative therapies. The research highlighted at this meeting and the new collaborations formed between colleagues will undoubtedly contribute to these efforts.

## Author Contributions

ES, MM-L, and BL-C served as co-organizers of the BMA2020 meeting and worked together to develop the conference report herein. All authors contributed to the article and approved the submitted version.

## Funding

The authors declare that this research conference received funding from Radius Heath Inc., Kubtec, The Jackson Laboratory, Progen, Washington University Musculoskeletal Research Center, and BioQuant. The funders were not involved in the conference design, the writing of this article or the decision to submit it for publication.

## Conflict of Interest

The authors declare that the research was conducted in the absence of any commercial or financial relationships that could be construed as a potential conflict of interest.

The reviewer WC declared a past collaboration with one of the authors ES to the handling editor.
