# Supplementary material for: Report From the 6th International Meeting on Bone Marrow Adiposity (BMA2020)
Source: Front Endocrinol (Lausanne). 2021 Jul 16;12:712088. doi: 10.3389/fendo.2021.712088 (PMC8323480; doi:10.3389/fendo.2021.712088)
Supplement: Supplementary file 1 [file Table_1.docx]

**Supplemental Table 1.** Plenary sessions, presenters, and talk titles.

| **Session** | **Presenter** | **Title** |
| --- | --- | --- |
| Keynote | Ormond MacDougald | Keynote: Bone marrow adipose tissue (BMAT) biology, endocrine physiology and metabolism – unique and common traits with peripheral adipose tissue depots |
| Session I: Bone Marrow Adipose Tissue, Aging, and Skeletal Homeostasis | Sundeep Khosla | Plenary Talk 1: BMAT, aging, senolytics and skeletal health |
|  | Anuj Sharma | Short Talk 1: Sex differences in skeletal development and maintenance mediated by the osteoblastic glucocorticoid receptor |
|  | Abhishek Chandra | Short Talk 2: Role of Senescence in Age- and Radiation- Associated Bone Marrow Adiposity |
|  | Thomas H. Ambrosi | Short Talk 3: Loss of skeletal stem cell diversity drives a pro-inflammatory, pro-adipogenic bone marrow niche |
|  | Vagelis Rinotas | Short Talk 4: Interplay between Bone Marrow Adiposity and bone loss in TgRANKL osteoporotic models |
| Session II: Environmental Regulation of Bone and Marrow Adipose Tissue | Pouneh Fazeli | Plenary Talk 2: BMAT and bone in states of obesity and malnutrition |
|  | Janet Rubin | Plenary Talk 3: Exercise, BMAT, and bone |
|  | Piotr Czernik | Short Talk 5: Reconstitution of the host holobiont acutely increases bone growth and marrow adiposity of the gnotobiotic rat |
| Session III: Endocrine Regulation of Bone Marrow Adipose Tissue in Health and Disease | Clifford Rosen | Plenary Talk 4: PTH/Endocrine regulation of Bone Marrow Adiposity |
|  | Nikki Aaron | Short Talk 6: Adipsin as a novel adipokine regulating bone remodeling |
|  | Sudipta Baroi | Short Talk 7: PPARG in Osteocytes Regulates Sclerostin Expression, Bone Mass and Marrow Adiposity |
|  | Li Chen | Short Talk 8: A secreted factor from bone that regulates bone and fat formation and metabolism balance |
| Session IV: Advanced Methods for Clinical and Pre-Clinical Assessment of Bone Marrow Adiposity and Skeletal Health | Gustavo Duque | Plenary Talk 5: Assessing lipotoxicity in bone |
|  | Greet Kerckhofs | Plenary Talk 6: Novel pre-clinical contrast agents for 3D imaging of BMAT |
|  | Kisoo Pahk | Short Talk 9: Visceral Fat Metabolic Activity Evaluated by 18F-FDG PET/CT Predicts Osteoporosis in Healthy Postmenopausal Korean Women |
|  | Josefine Tratwal | Short Talk 10: An injectable 3D tissue-engineered model of bone marrow adipogenesis and hematopoiesis |
| Session V: Bone Marrow Adipose Tissue, Cancer, and Hematopoiesis | Olaia Naiveras | Plenary Talk 7: BMAT, bone marrow reconstitution, and hematopoiesis |
|  | Izabela Podgorski | Plenary Talk 8: BMAT and cancer metastasis to bone |
|  | Emma Morris | Short Talk 11: The PPAR-gamma antagonist Bisphenol A diglycidyl ether induces myeloma cell death in vitro and in vivo via a PPAR-gamma independent mechanism |
|  | Sonia Severin | Short Talk 12: A close relationship between adipocytes and megakaryocytes: a link with obesity |
|  | Mariah Farrell | Short Talk 13: Bone marrow adipocytes induce multiple myeloma cell adipomimicry and dexamethasone resistance |
| Session VI: Bone Marrow Adipose Tissue Origins and Maturation | Moustapha Kassem | Plenary Talk 9: Secreted factors which regulate stem cell commitment to BMA and bone fragility |
|  | Russell Turner | Short Talk 14: Role of Kit Signaling in Maturation of Bone Marrow Adipocytes in Mouse Long Bones |
|  | Leilei Zhong | Short Talk 15: Single cell transcriptomics identifies a unique adipose lineage cell population that regulates bone marrow environment |
|  | Xiao Zhang | Short Talk 16: Fat in a fatless mouse: a novel origin of maladapted bone marrow adipocytes with age and disease |
| Session VII: Bone Marrow Adipocyte Differentiation, Metabolism and the Skeletal Niche | Courtney Karner | Plenary Talk 10: Novel metabolic pathways which mediate BMA progenitor differentiation |
|  | Ziru Li | Short Talk 17: Cellular interactions in the bone marrow niche evaluated with a bone marrow adipocyte-specific knockout mouse model |
|  | Ayyoub Salmi | Short Talk 18: An integrative bioinformatics approach to dissect adipocyte-induced transdifferentiation of osteoblast |
|  | Amit Chougule | Short Talk 19: PPARα in osteocytes regulates bone marrow adipose tissue development |
|  | Wei Yu | Short Talk 20: Bone marrow adipogenic lineage precursors (MALPs) promote osteoclastogenesis in bone remodeling and pathologic bone loss |
