# Supplementary material for: Report From the 6th International Meeting on Bone Marrow Adiposity (BMA2020)
Source: Front Endocrinol (Lausanne). 2021 Jul 16;12:712088. doi: 10.3389/fendo.2021.712088 (PMC8323480; doi:10.3389/fendo.2021.712088)
Supplement: Supplementary file 2 [file Table_2.docx]

**Supplemental Table 2.** Poster presenters and titles.

| **Poster Number** | **Presenter** | **Title** |
| --- | --- | --- |
| 1 | Kenneth T. Lewis | Bone Marrow Adipocytes Arise Predominantly from Adiponectin-Expressing Precursors During Caloric-Restriction Induced Bone Marrow Adipose Tissue Expansion. |
| 2 | Xiao Zhang | Neural contributions to leptin-mediated bone marrow adipocyte catabolism |
| 3 | Victoria DeMambro | Prrx1-CreAlplfl/fl mice, a model for further investigations into the pathophysiological changes in hypophosphatemia |
| 4 | Dalia Ayesh Hafez Ali | High fat diet (HFD)-induced obesity augments the deleterious effects of Estrogen deficiency in bone and bone marrow micro-environment. Evidence from ovariectomized mice. |
| 5 | Michaela Tencerova | The effect of novel “PPARγ-sparing” thiazolidinedione on bone and metabolic phenotype in high fat diet-fed mice. |
| 6 | Jingruo Zhang | Modulation of Bone Marrow Mesenchymal Stem Cell Differentiation by Osteocytic Connexin Hemichannels |
| 7 | Zengdi Zhang | Metabolic control of the bone-fat balance via O-GlcNAc signaling |
| 8 | Katja Wegener | Characterizing the Hyaluronan-rich matrix in bone marrow adipose tissue |
| 9 | Benoit von der Weid | An investigation of human stromal cell heterogeneity in post-chemotherapy marrow by scRNA-sequencing of healthy and leukemic marrow aspirates |
| 10 | Ahmed Al Saedi | Lipid Signaling Mediators regulates myogenesis with ageing |
| 11 | Rebecca Schill | Determining the Role of the Glucocorticoid Receptor on Bone Marrow Adipose Tissue Expansion During Calorie Restriction |
| 12 | Priyanka Kushwaha | Maternal high-fat diet intake during gestation and lactation induces long-lasting defects in bone micro architecture in rat offspring through enhanced osteoclastogenesis |
| 13 | Andrea Lovdel | Investigating the association between ageing, glucocorticoids, bone marrow adiposity expansion and bone loss |
| 14 | Kathleen Becker | C3H substrain variation results in decreased bone density and fat mass in C3H/HeJ mice |
| 15 | Vanessa Sherk | Independent effects of estrogen and follicle stimulating hormone on marrow cell populations identified by single cell RNA-Seq analysis |
| 16 | Ho Jun Kang | Lactobacillus reuteri prevents Early Stages of Glucocorticoid-induced Avascular Necrosis of Femoral Head. |
| 17 | Abbas Jafari | CD10: A novel marker of bone marrow adipocyte progenitors |
| 18 | Siddaraju Boregowda | Reduced A2M Expression in Skeletal Stem Cells Promotes Marrow Adiposity During Aging |
| 19 | Julio Carballido-Gamio | Assessment of bone mineral density, cortical bone thickness and bone marrow fat at the proximal femur in HIV-infected subjects |
| 20 | Yuhei Uda | PTH protects osteocytes from oxidative stress-induced death and senescence |
| 21 | Trisha Hue | Longitudinal Evaluation of Saturated and Unsaturated Vertebral Marrow Adipose Tissue (MAT) Over 3 Years in Older Men and Women |
| 22 | Nico Sollmann | Vertebral bone marrow heterogeneity using texture analysis of chemical shift encoding-based water-fat MRI: variations in age, sex, and anatomical location |
| 23 | Erica Clinkenbeard | Differential iron requirements of osteoblast and adipocyte differentiation |
| 24 | Heather Fairfield | Myeloma-modified adipocytes exhibit metabolic dysfunction and a senescence-associated secretory phenotype (SASP) |
| 25 | Jean-François Budzik | BONE PERFUSION AND ADIPOSITY BEYOND THE NECROTIC ZONE IN FEMORAL HEAD OSTEONECROSIS: A QUANTITATIVE MRI STUDY |
| 26 | Shuang Zhang | ENDOTHELIAL CELL PHYSIOLOGY IN A MICROFLUIDIC DEVICE AND THEIR RESPONSE TO MESENCHYMAL STROMAL CELLS |
| 27 | Rita Sarkis | MarrowQuant in human trephine biopsies: a Digital Pathology tool for interrogating bone marrow architecture in acute myeloid leukemia |
| 28 | Ahmed Al Saedi | 1,25(OH)D3 ameliorates palmitate-induced lipotoxicity in human primary osteoblasts leading to improved viability and function |
| 29 | Laura Entz | Characterization of the extracellular matrix of Bone Marrow Adipocytes in hyperglycemic condition in vitro |
| 30 | Jason A Horton | Oxylipins Mediate Radiation-induced Marrow Adipose Tissue Expansion |
| 31 | Viktorija Avilkina | HOW THE SEVERITY LEVEL OF ENERGY DEFICIT IN MICE MODEL AFFECTS THE BONE MARROW ADIPOSITY, BONE QUALITY AND BONE MARROW STROMAL CELLS DIFFERENTIATION? |
| 32 | Gina Woods | Saturated and Unsaturated Bone Marrow Lipids Have Distinct Effects on Bone Density and Fracture Risk in Older Adults |
| 33 | Nico Sollmann | Local BMD, VAT, and SAT measurements in routine computed tomography – which parameter predicts osteoporotic vertebral fractures best? |
| 34 | Lutian Yao | Single cell analysis reveals transient expansion of marrow adipogenic lineage precursors as the mechanism for bone marrow recovery after radiation |
| 35 | Karen De Samblancx | aE-catenin Deletion in Skeletal Stem and Progenitor Cells (SSPCs) Increases their Adipogenic Potential and Protects Against Diet- or Age-induced Obesity and Hyperglycemia |
