# Supplementary material for: Report From the 6th International Meeting on Bone Marrow Adiposity (BMA2020)
Source: Front Endocrinol (Lausanne). 2021 Jul 16;12:712088. doi: 10.3389/fendo.2021.712088 (PMC8323480; doi:10.3389/fendo.2021.712088)
Supplement: Supplementary file 3 [file Table_3.docx]

**Supplemental Table 3.** Scientific Awards.

| Award category | Place | Recipient | Recipient career stage | Recipient institution |
| --- | --- | --- | --- | --- |
| Best basic/translational research abstract | 1 (tie) | 1. Ziru Li 2. Xiao Zhang | 1. Postdoctoral fellow 2. PhD student | 1. University of Michigan 2. Washington University in St. Louis |
|  | 2 (tie) | 1. Leilei Zhong 2. Michaela Reagan 3. Russel Turner | 1. Postdoctoral fellow 2. Assistant Professor 3. Professor | 1. University of Pennsylvania 2. Maine Medical Center 3. Oregon State University |
|  | 3 (tie) | 1. Wei Yu 2. Lutian Yao | 1. Visiting scholar 2. Postdoctoral fellow | 1. University of Pennsylvania 2. University of Pennsylvania |
| Best clinical research abstract | 1 | Gina Woods | Associate Professor | University of California San Diego |
|  | 2 | Kisoo Pahk | Postdoctoral fellow | Korea University Anam Hospital |
|  | 3 | Julio Carbadillo-Gamio | Assistant Professor | University of Colorado |
| Best short talks |  | 1. Thomas Ambrosi 2. Sonia Severin 3. Sudipta Baroi 4. Nikki Aaron 5. Amit Chougule | 1. Postdoctoral fellow 2. Academic researcher 3. PhD student 4. PhD student 5. Postdoctoral fellow | 1. Stanford University 2. Inserm 3. University of Toledo 4. Columbia University 5. University of Toledo |
| Best poster presentations |  | 1. Abbas Jafari 2. Xiao Zhang 3. Karen de Samblancx 4. Rebecca Schill | 1. Assistant Professor 2. PhD student 3. PhD student 4. Postdoctoral fellow | 1. University of Copenhagan 2. Washington University in St. Louis 3. KU Leuven 4. University of Michigan |
| Best poster presentation – audience choice |  | Kenneth Lewis | Postdoctoral fellow | University of Michigan |
